# Intravascular Imaging for Facilitated Coronary Interventions in DES Era

**DOI:** 10.3390/jcdd13010038

**Published:** 2026-01-09

**Authors:** Gönül Zeren, Eren Ozan Bakır, Vincenzo Tufaro, Ayşe Nur Özkaya, Tingquan Zhou, Sotiris Kyriakou, Jae-Geun Lee, Yoshinobu Onuma, Patrick W. Serruys, Christos V. Bourantas

**Affiliations:** 1 Centre for Cardiovascular Medicine and Devices, William Harvey Research Institute, Queen Mary University, London E1 4NS, UK; gonulzeren@hotmail.com (G.Z.); eren.ozan.bakir@gmail.com (E.O.B.); dr.aysenur.ozkaya@gmail.com (A.N.Ö.); tingquanzhou2023@gmail.com (T.Z.); tedljg@gmail.com (J.-G.L.); 2 Department of Cardiology, Siyami Ersek Training and Research Hospital, University of Health Sciences, Istanbul 34668, Turkey; 3 Department of Cardiology, Barts Heart Centre, Barts Health NHS Trust, London EC1A 7BE, UK; vincenzotufaro@hotmail.it (V.T.); ssotos3201@gmail.com (S.K.); 4 Department of Cardiology, National University of Ireland, H91 TK33 Galway, Ireland; yoshinobuonuma@gmail.com (Y.O.); patrick.w.j.c.serruys@gmail.com (P.W.S.); 5 Faculty of Medicine, National Heart & Lung Institute, Imperial College, London SW7 2AZ, UK

**Keywords:** intravascular imaging, intravascular ultrasound, optical coherence tomography, percutaneous coronary intervention, drug-eluting stents

## Abstract

Intravascular imaging (IVI) was introduced 35 years ago to assess coronary artery pathology and plaque vulnerability. However, from its first applications it became apparent that it can also be useful in percutaneous coronary intervention (PCI) planning and optimizing PCI results. In the early days of PCI, IVI was used to examine the efficacy of emerging endovascular devices and the vessel wall response to therapy, while in the drug-eluting stent (DES) era, IVI was used to guide DES implantation and assess final results post-intervention. The first studies assessing the role of IVI in guiding PCI with DES have failed to demonstrate a prognostic benefit for the use of IVI; however, more recent large-scale randomized trials have underscored its value in this setting. IVI, with its high resolution, allows optimal stent sizing, prompt identification and correction of common causes of stent failure, and it has been shown that it improves outcomes in complex procedures. This review summarizes the evidence supporting the role of IVI in PCI planning in DES era, synopsizes the studies that have highlighted the value of IVI in predicting stent failure, discusses the limitations of the first randomized trials that failed to demonstrate a prognostic benefit from its use, and presents the results of the more recent large-scale outcome studies that underscored its role in complex PCI planning.

## 1. Introduction

Intravascular imaging (IVI) was introduced in the 1990s to assess coronary artery morphology in vivo and predict plaques that are prone to progress and cause cardiovascular events [1]. However, from its first applications it became apparent that it also enables evaluation of vessel wall response following percutaneous coronary intervention (PCI), enabling evaluation of the performance of different treatment strategies [2]. Both intravascular ultrasound (IVUS)—the first IVI modality introduced in clinical practice—and also optical coherence tomography (OCT) have played an indispensable role in the maturation of PCI [3]. In the early days of PCI, IVUS was used to examine the mode of action of plain old balloon angioplasty (POBA) and showed that lumen enlargement following POBA is due to plaque dissection, vessel wall remodelling and plaque redistribution, while in atherectomy the increase in the lumen area is mainly due to a reduction in plaque area [4,5,6]. Subsequent studies using serial IVI shed light onto the causes of restenosis, showing that this is due to intimal proliferation and negative remodelling [7,8]. The latter challenge was addressed with the introduction of the bare metal stents that reduced the incidence of restenosis; nevertheless, their implantation was associated with a risk of stent thrombosis (ST) [9]. IVI studies have been proven useful in understanding the mechanism of in-stent restenosis (ISR), showing that this is due to neointimal proliferation, and also in revealing predictors of ST [10,11]. The development of the DES that incorporated an antiproliferative drug in their metallic scaffold and a polymer to control its delivery changed the landscape of coronary interventions, as this minimized the risk of ISR and enabled treatment of complex lesions in patients. IVI studies have been extensively performed in the first years of DES to assess the efficacy of these devices but also to investigate the mechanism of late ST that was noted in the first-generation DES and was attributed to an inflammatory response of the vessel wall to the polymer used to control drug kinetics [12,13]. The updated revision of DES with thinner struts that were polymer-free or had a biocompatible or biodegradable polymer and more effective antiproliferative drugs improved the safety of DES and reduced the event rate [14]. In this setting, the first IVI studies have failed to demonstrate a prognostic benefit from the use of IVUS or OCT during PCI. However, more recent studies including a large number of patients undergoing complex PCI have underscored the value of both modalities in guiding revascularization. This review aims to provide an overview of the evidence that support the use of IVI in PCI planning. It summarizes the findings of studies that have highlighted the role of IVI in detecting suboptimal PCI results, discusses the results of the first randomized control studies (RCT) in the DES era that have failed to demonstrate a prognostic benefit from the use of IVI, and presents the more recent trials and metanalyses that have highlighted the role of IVI during DES implantation.

## 2. Intravascular Imaging Studies Identifying Predictors of Cardiovascular Events

**Figure 1:**
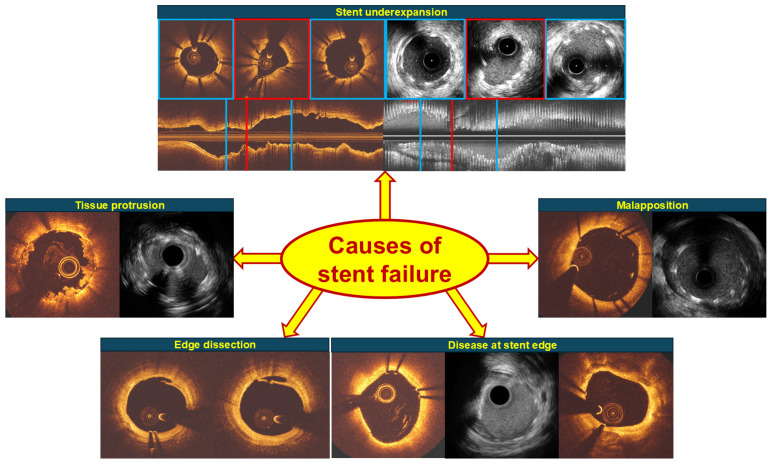
Post-PCI features associated with a higher incidence of ISR and ST.

IVI studies have provided unique insights about the mechanisms of ISR and ST in DES. Stent underexpansion, disease at the stent edge and large edge dissections, thrombus or tissue protrusion and malapposition have been found to be predictors of stent failure and constitute targets for therapy today (Figure 1).

### 2.1. Underexpansion

A small stent area has been found to be a predictor of stent failure in several studies. In the left main stem (LMS) PCI, an IVUS study in an Asian population has shown that a small minimum stent area (MSA, cutoffs: in proximal LMS < 8.2 mm^2^, in distal LMS < 7.2 mm^2^, in proximal left anterior descending < 6.3 mm^2^ and in proximal left circumflex artery < 5.3 mm^2^) was a predictor of stent failure at 9 months follow-up [15]. These cutoffs were larger in the EXCEL trial (distal bifurcation lesions: 9.8 ± 2.3 mm^2^; ostial/shaft lesions: 10.4 ± 2.5 mm^2^) where a MSA of 4.4–8.7 mm^2^ was related with a higher incidence of the combined endpoint of death, myocardial infarction (MI) or stroke at 3 years follow-up [16]. In non-LMS lesions, several IVUS studies have shown that a MSA of <5.5 mm^2^ assessed by IVUS was a predictor of ISR [17,18], whereas in OCT this cutoff has been found to be <4.5 mm^2^ in the CLI-OPCI II registry and 4 mm^2^ in the ILLUMIEN IV study, as the risk of target-vessel failure (TVF) increased exponentially in lesions with a MSA below this cutoff [19,20].

Stent underexpansion, defined as the ratio of the MSA vs. a reference lumen area, has been found in several studies to be a predictor of ST, but there is limited evidence supporting its role in predicting restenosis [21,22,23]. Today, based on the European Association Percutaneous Coronary Intervention consensus document, a cutoff of stent expansion of 80%, defined as the ratio of the MSA x 100 vs. the mean reference lumen area, has been used to define optimal stent expansion [24].

### 2.2. Edge Dissection and Plaque at Stent Edge

Several studies have shown that large edge dissections are predictors of ST, while a post hoc analysis of the ILUMIEN IV study has shown that edge dissection is associated with a higher incidence of ischemia-driven target-lesion revascularization (TLR) [19,25,26].

In addition, disease at the stent edge has been found to be a predictor of edge restenosis. In the study by Kang et al., a small reference lumen area (<5.7 mm^2^) and the presence of a large plaque burden (>54.5%) at the reference segment on IVUS were predictors of edge restenosis [27]. Similar were the findings of Liu et al., who showed that a plaque burden cutoff of 47.1% measured by IVUS was a predictor of edge restenosis in paclitaxel eluting stents at 9 months [28]. In addition, in the study of Ino et al. a small minimum reference lumen area (≤4.1 mm^2^) assessed by OCT and the presence of lipid (lipid arc ≥ 185°) at the edge of the stent were predictors of angiographic restenosis at 9 months [29]. Finally, in the CLI-OPCI registry, a reference lumen area < 4.5 mm^2^ on OCT was related with worse prognosis [30].

Considering the above studies, it is recommended today to avoid landing stents in segments with plaque burden > 50% and lipid arc > 180°. Moreover, treatment of edge dissection with an additional stent is advised when these have a length > 2 mm or an arc > 60° [24].

### 2.3. Tissue or Thrombus Protrusion

Intra-stent tissue or thrombus protrusion has been associated with a high event rate. It has been argued that this is due to a rapid endothelization of the protruded mass leading to aggressive ISR and stent failure [31,32,33]. Moreover, protruding thrombus can cause flow obstruction and activate the coagulation cascade, leading to thrombus formation and a higher risk of ST [25,34,35]. Therefore, it is recommended in cases of intrastent large thrombus or tissue protrusion to perform further intervention and optimize PCI results.

### 2.4. Malapposition

The prognostic implications of strut malapposition are less well established. Numerous prospective studies performing IVUS or OCT imaging at the end of the procedure have failed to show an association between strut malapposition and ST [25,33,36]. It was only recently that two reports demonstrated a higher ST rate in patients with malapposed struts at OCT imaging post PCI [35,37]. The link between strut malapposition and ST has also been confirmed in registries recruiting patients that had ST and OCT imaging at the time of their event [21,22,23,35,37,38,39,40,41]. Considering this evidence, it is recommended to perform additional post dilatation in cases of extensive malapposition (length ≥ 1 mm and axial distance ≥ 0.4 mm) [24].

The ability of IVI to identify features associated with worse outcomes and predict prognosis renders it not only useful in risk stratification but also an indispensable tool for optimizing PCI results and improving clinical outcomes. The evidence supporting the prognostic benefit of IVI in the DES era is presented below.

## 3. Studies on Intravascular Imaging-Guided PCI: From Past to Present

### 3.1. Evidence Supporting the Use of IVUS in the Guiding DES Implantation

The first observational studies in DES have failed to demonstrate better outcomes from the use of IVI and, in particular, of IVUS. Some of these studies were limited by the small number of patients included, while the large observational studies provided conflicting findings. In a propensity matched study including 1768 patients, Roy et al. failed to demonstrate a reduction in the major adverse cardiovascular events (MACE) rate at 12 months follow-up in the IVUS cohort, although there was a lower incidence of ST in this group compared to the angiography-guided arm [42]. Similar were the findings of the Excellent study including 1421 patients, while Hur et al., in a study including 8371 patients implanted with a 1st-generation DES, showed no difference in the MACE rate at 3 years follow-up in the IVUS and angiography-guide group but a lower mortality in the IVUS arm [43,44]. In the KAMIR registry that recruited 14,329 patients with an acute MI, and in the MATRIX registry which recruited 1504 patients implanted with a 1st generation DES, there was no difference in the MACE rate at 12 months in the IVUS-guided and the angiography-guided PCI, although there was a lower incidence of MI in the IVUS arm [45,46]. The ADAPT-DES registry was the first large registry that underscored the value of IVUS imaging in guiding PCI, showing that its use reduced the incidence of MI and ST at 1 year follow-up, a benefit that persisted up to 2 years [47,48]. In line with these findings, the first studies in bifurcation lesions demonstrated a lower incidence of the combined endpoint of death or MI at 2 and 4 years follow-up, findings that were also confirmed by Chen et al. showing that, in bifurcation lesions treated with a 2-stent strategy, IVUS reduced the incidence of ST and MI at 12 months follow-up [49,50,51].

However, the first RCTs comparing outcomes following IVUS- and angiography-guided PCI failed to demonstrate a prognostic benefit from the use of IVUS. In the HOME-DES trial including 210 patients with complex lesions randomized to IVUS-guided or angiography-guided DES therapy, there was no difference in outcomes in the two cohorts [52]. Similar were the findings of the AVIO study that included 284 patients with complex lesions and of the RESET trial which recruited 543 patients with long lesions who were randomized to IVUS- and angiography-guided PCI; both studies showed a similar MACE rate in the two groups [53,54]. Finally, the AIR-CTO study that included 230 patients was the first RCT that examined the value of IVUS imaging in guiding revascularization in chronic total occlusions (CTO) and showed a smaller late lumen loss in the IVUS group at 1 year follow-up; however, there was no difference in the incidence of MACE in the studied groups at 2 years [55].

While the first RCTs failed to provide evidence to support a prognostic value from the use of IVUS in DES implantation, cumulative evidence coming from registries continued to show better outcomes from the use of IVI [56,57,58]. The discrepancy between the findings of RCTs and registries should be attributed to the fact that the first trials included a small number of patients and, therefore, were unable to demonstrate a prognostic benefit from the use of IVUS and to the fact that most of the studies did not have pre-specified criteria for optimal stent implantation in the IVUS arm; this resulted in operator-dependent stent deployment in this group and limited the benefit of IVUS guidance in stent optimization.

The first RCT showing a lower MACE rate from the use of IVUS was the study of Tan et al. in 2015 [59]. In this study, the investigators included 123 patients with unprotected LMS disease who were randomized to IVUS- and angiography-guided DES implantation. The investigators showed a lower incidence of MACE at 2 years in the IVUS group attributed mainly to a lower rate of TLR. However, this was a single-centre study that included a small number of patients; in addition, the number of the events reported was too small to allow drawing safe conclusions. The CTO-IVUS study published a few months later was the first RCT that underscored the value of IVUS in CTOs, showing that its use is associated with a 64% reduction in the incidence of cardiac death, MI and TLR; conversely, the IVUS-XPL study, the largest RCT conducted until that point that included 1400 patients with long lesions, demonstrated that IVUS-guided PCI was related to a lower MACE rate at 1 year compared to angiography-guided revascularization (2.9% vs. 5.8%, *p* = 0.007), a prognostic benefit that was also maintained at 5 years follow-up [60,61,62].

The ULTIMATE study was the first prospective all-comer RCT comparing outcomes following IVUS- and angiography-guided revascularization. The authors randomized 1448 patients and demonstrated a lower incidence of target-vessel failure (TVF)—that included the composite of cardiac death, target-vessel MI and clinically driven target-vessel revascularization—in the IVUS arm at 1 year [63].

**Figure 2:**
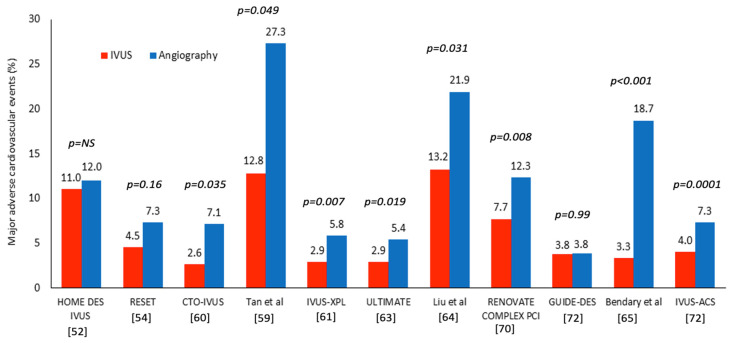
RCTs comparing outcomes in patients undergoing IVUS-guided and angiography-guided PCI [52,54,59,60,61,63,64,65,70,72].

Since then, numerous RCTs have been published that provided consistent results. Two studies reported by Liu et al. and Bendary et al., including 336 and 181 patients with LMS disease, randomized patients in IVUS- and angiography-guided PCI and demonstrated a lower MACE rate at 1 year in the IVUS arm (13.2% vs. 21.9%, *p* = 0.031 and 3.3% vs. 18.7%, *p* < 0.001, respectively) [64,65]. These findings are in line with large registry data supporting the use of IVI in this setting and contradict the results from the post hoc analysis of the NOBLE study where IVUS use was not associated with a better prognosis [66,67,68,69]. Moreover, the RENOVATE-COMPLEX trial that assigned 1639 patients with complex coronary artery lesions at a 2:1 ratio to IVUS- (73.3%) or OCT- (25.5%) and angiography-guided revascularization demonstrated that IVI guidance was associated with a lower incidence of TVF at 2.1 years of follow-up (7.7% vs. 12.3%) [70]. Similar were the findings of the IVUS-ACS study that randomized 3505 patients with acute coronary syndrome (ACS) to IVUS- or angiography-guided revascularization and reported a 45% reduction in the incidence of TVF at 1 year (4.0% vs. 7.3%), driven mainly by a reduction in the incidence of MI and TLR [71]. Conversely, the GUIDE DES trial that randomized 1528 patients to IVUS- or quantitative coronary angiography-guided PCI failed to demonstrate a difference in the primary endpoint consisting of cardiac death, target-vessel MI and ischemia-TRL at 12 months follow-up (3.80% vs. 3.81%) [72]. However, in this non-inferiority study the number of the events reported was smaller to the expected event rate (8%) in the power calculation; therefore, the results of this analysis should be interpreted with caution (Figure 2, Table 1).

### 3.2. Evidence Supporting the Use of OCT in Guiding DES Implantation

The first studies comparing outcomes in patients undergoing angiography- and OCT-guided PCI have failed to demonstrate a prognostic benefit in the OCT arm. The DOCTORS study was the first to randomize 240 patients with a non-ST-elevation MI to angiography- or OCT-guided PCI and had the post-procedural fractional flow reserve (FFR) values as a primary endpoint. The investigators found a higher FFR in the OCT arm (0.94 ± 0.04 vs. 0.92 ± 0.05, *p* = 0.005); however, there was no difference in the outcomes in the two groups at 6 months [73]. In line with these findings, the ROBUST study that randomized patients with a ST-elevation MI to OCT- or angiography-guided revascularization showed a lower in-segment area stenosis (6% vs. 18%; *p* = 0.0002) but no prognostic benefit from the use of OCT at 9 months follow-up (3% vs. 2%; *p* = 0.87) [74]. Finally, the ILUMIEN III and the i-Sight RCTs that were conducted to examine procedural outcomes in patients undergoing IVUS-, OCT- or angiography-guided revascularization reported no difference in the prognosis in the three groups; however the number of the included patients and the events were too small to allow us to draw safe conclusions [75,76].

The Pan London PCI registry was the first report that underscored the prognostic value of OCT in planning revascularization. The investigators analyzed data from 87,166 patients undergoing PCI in London hospitals and showed a lower incidence of in-hospital MACE in patients undergoing IVUS- or OCT-guided revascularization compared to the angiography arm. The mortality rate was lower in the OCT group (7.7%) compared to the IVUS- (12.2%) or angiography-guided cohort (15.7%, *p* < 0.0001). Propensity matched analysis and showed a lower mortality rate in the OCT compared to the angiography arm (9.60% vs. 16.80%; *p* < 0.001) but no difference between OCT- and IVUS-guided PCI (8.96% vs. 10.20%; *p* = 0.12) [77].

Similar were the findings of the RENOVATE-COMPLEX trial which included 1639 patients with complex coronary artery disease undergoing IVI- (with IVUS or OCT) and angiography-guided PCI. A lower TVF rate was reported in the IVI arm (7.7% vs. 12.3%, *p* = 0.008); forest plot analysis showed a lower event rate in both group of patients undergoing IVUS- or OCT-guided PCI compared to those listed for angiography-guided revascularization (hazard ratio: 0.66, 95% confidence interval: 0.46–0.95 for the IVUS-guided group and 0.47, 95% confidence interval: 0.27–0.83 for the OCT cohort) [70].

The ILLUMEN IV was the first large, appropriately powered RCT that compared outcomes in patients undergoing OCT- and angiography-guided PCI. In this study, 2487 patients with either diabetes or complex disease including long lesions, bifurcation lesions, CTOs, ISR and calcified lesions were randomized to OCT- or angiography-guided revascularization. PCI was performed by experienced operators in OCT, and, in contrast to previous studies, strict criteria were proposed to define optimal PCI results in the OCT arm. Patients undergoing PCI under OCT guidance had a larger MSA post PCI compared to those treated under angiographic guidance (5.72 ± 2.04 mm^2^ vs. 5.36 ± 1.8 mm^2^, *p* < 0.001); however, at 2 years follow-up, OCT-guided PCI was not associated with a lower incidence of TVF—the primary clinical endpoint of the study which included cardiac death, target-vessel MI and ischemia-driven target-vessel revascularization (TVR)–(7.4% vs. 8.2%, *p* = 0.45). The lack of prognostic benefit from the use of OCT was attributed by the investigators to the low TVR in the angiography arm, and this was considered related to the fact that patient follow-up was performed during the COVID-19 pandemic [78].

Different were the findings of the OCCUPI study that recruited 1604 patients undergoing PCI in complex lesions who were randomized at a 1:1 ratio to OCT- or angiography-guided revascularization. During a follow-up period of 1 year, the primary endpoint of the study, which consisted of the composite of cardiac death, MI, stent thrombosis, or ischemia-driven TVR, occurred in 96 patients; a higher event rate was noted in the angiography compared to the OCT group (5% vs. 7%, *p* = 0.023) [79].

**Figure 3:**
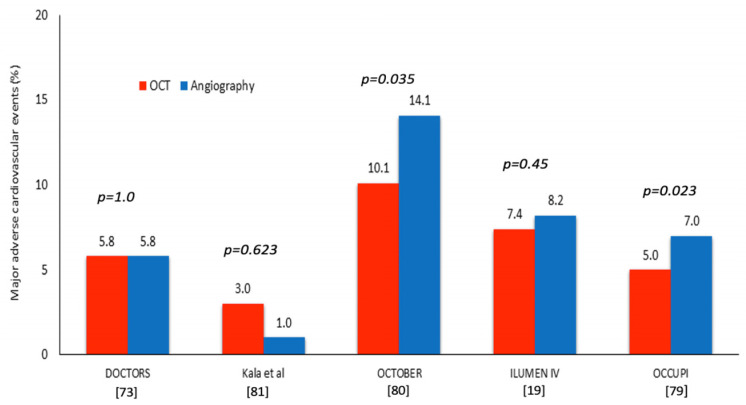
RCTs comparing outcomes in patients undergoing OCT-guided and angiography-guided PCI [19,73,79,80,81].

Similar were the findings of the OCTOBER study, the only OCT study focusing on bifurcation lesions. The investigators recruited 1201 patients undergoing PCI in a bifurcation lesion that were randomized at a 1:1 ratio to OCT- or angiography-guided revascularization. At 2 years follow-up, a higher MACE—defined as cardiac death, target-lesion MI, or ischemia-driven TLR—rate was noted in the angiography arm compared to the OCT group (14.1% vs. 10.1%, *p* = 0.035) [80] (Figure 3, Table 2).

### 3.3. IVUS or OCT for Guiding PCI

**Figure 4:**
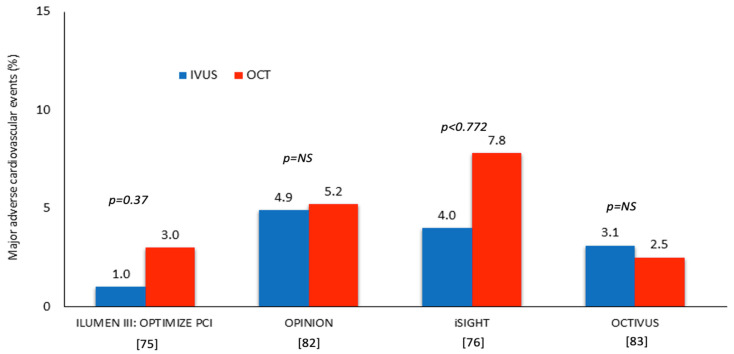
RCTs comparing outcomes in patients undergoing IVUS-guided and OCT-guided PCI [75,76,82,83].

Several studies have recently compared clinical outcomes in patients undergoing IVUS- and OCT-guided PCI (Figure 4). In the OPINION study, 829 patients with de novo coronary artery lesions listed for PCI were randomized to OCT- or IVUS-guided intervention. At 1 year follow-up, the primary endpoint consisted of the composite of cardiac death, target-vessel related MI, and ischemia-driven TVR which occurred in 5.2% of the patients in the OCT and in 4.9% of the patients in the IVUS arm (*p*_non-inferiority_ = 0.042). A major limitation of this study was the lower event rate in the IVUS arm than the estimated in the power calculation (9%) that may have rendered the study underpowered for the primary endpoint [82].

The OCTIVUS RCT randomized 2008 patients with obstructive coronary artery disease to IVUS- and OCT-guided PCI at a 1:1 ratio; at 1 year follow-up, the primary endpoint of TVF occurred in 2.5% in the OCT and in 3.1% in the IVUS group (*p*_non-inferiority_ < 0.001). However, similarly to the OPINION study, this study had insufficient statistical power for the primary endpoint, as the estimated event rate in the IVUS arm in the power calculation was significantly higher than the actual event rate (8% vs. 3.1%) [83].

This limitation was overcome by recently published network metanalyses comparing outcomes in OCT-, IVUS- and angiography-guided PCI. All have demonstrated that IVI resulted in a significant reduction in the MACE rate including hard clinical endpoints such as all-cause death, cardiac death of any MI or target-vessel MI and ST [84,85,86]. In these studies, both IVUS and OCT appear superior to angiography in improving outcomes. Considering the above evidence, both IVUS and OCT have IA indication in the recently published European Society of Cardiology and the American College of Cardiology guidelines for the treatment of patients with complex lesions [87,88] (Figure 4, Table 3).

## 4. Discussion

Although the first RCTs have failed to demonstrate a prognostic benefit from the use of IVI in the DES era, recent evidence has underscored its role in PCI planning. Cumulative data have shown that IVUS- or OCT-guided DES implantation is associated with a prognostic benefit that compares favourably with the prognostic benefit provided by established pharmacotherapies such as high-dose statins or PCSK9 inhibitors [89,90,91,92]. Based on the published literature, IVI should be considered to guide revascularization in most of the clinical scenarios as there is evidence indicating a prognostic benefit from its use in LMS, bifurcation lesions, long lesions, calcified lesions, CTOs and in patients with an ACS [93].

However, despite this evidence, the use of IVI in clinical workflows is rather limited. In the United Kingdom, based on the British Cardiovascular Interventional Society audit data in 2024, only ≈25% of the PCIs were performed using IVI. This should be attributed to the fact that some of the interventional cardiologists are unable to interpret with confidence the IVUS or OCT images, due to the increased cost, the additional time required for IVI and the lack of evidence supporting a prognostic benefit of the use of IVI in drug-coated balloons (DCBs)—a DCB is used in ≈15% of the PCIs performed in the United Kingdom. Efforts have been made over recent years to overcome these barriers. Newer-generation IVUS catheters have an increased image acquisition rate and are pulled back at a faster speed to reduce the acquisition time, while fully automated software has been incorporated in IVUS and OCT systems to expedite the segmentation of the obtained images and guide DES implantation [94]. These developments, as well as the efforts that are made by the industry to train the interventional community, are expected to facilitate the broad use of IVI in PCI. In parallel, cumulative published data supports the use of IVI in planning revascularization with DCBs and in identifying lesions in patients with an ACS that can be safely left untreated [95,96,97]. Future studies as expected to explore the prognostic benefit of IVI in this setting; positive results will expand its applications and render it an indispensable tool in the catheterization laboratory.

## Figures and Tables

**Table 1:** RCTs comparing outcomes in patients having PCI under IVUS and angiographic guidance.

Study	Studied Groups	Number of Patients	Follow-Up (Months)	Lesion Type	Endpoints	Results
HOME DES IVUS [52]	IVUS vs. angiography-guided PCI	210 patients randomized at 1:1 ratio	18	Complex lesions type B_2_ and C, LMS or proximal LAD disease, small vessels (<2.5 mm), long lesions (>20 mm), ISR, patients with an ACS or ID-DM	- MACE including death, MI, TLR - ST	- There was no significant difference between groups in the incidence of MACE (11% vs. 12%; *p* = NS) or stent thrombosis (3.8 vs. 5.7%; *p* = NS)
AVIO [53]	IVUS vs. angiography-guided PCI	284 patients randomized at 1:1 ratio	24	Long lesions (>28 mm), CTOs, bifurcation lesions, small vessels (≤2.5 mm) and patients requiring four or more stents	- Post-procedural in-lesion MLD - MACE including any MI, cardiac death, TLR and TVR	- IVUS guidance was associated with a larger minimal lumen diameter (2.70 ± 0.46 mm vs. 2.51 ± 0.46 mm; *p* = 0.0002) than angiography-guided PCI - There was no difference in MACE at 24-month follow-up (7.0% vs. 8.5%, *p* = NS)
RESET [54]	IVUS vs. angiography-guided PCI	543 patients randomized at 1:1 ratio	12	De novo lesion requiring a stent ≥ 28 mm in length in a vessel with a distal reference diameter ≥ 2.5 mm	- MACE, including cardiovascular death, MI, ST and TVR	- There was no significant difference in the incidence of MACE, including CD, MI, ST, or TVR, between the IVUS-guided arm (4.5%) and the angiography-guided arm (7.3%, *p* = 0.16)
CTO-IVUS [60]	IVUS vs. angiography-guided revascularization	402 patients randomized at 1:1 ratio	12	CTO lesions	- Cardiac death - Secondary: MACE defined as the composite of cardiac death, MI, or TVR	- There was no difference in the incidence of cardiac death between groups (0% versus 1.0%; *p* = 0.16) - MACE rate was lower in patients having IVUS guided PCI than the angiography-guided group (2.6% vs. 7.1%; *p* = 0.035)
AIR-CTO [55]	IVUS vs. angiography-guided PCI	230 patients randomized at 1:1 ratio	24	CTO lesions that had been successfully recanalized	- In-stent LLL - Secondary: All-cause death, cardiac death, MI, ISR, TLR and TVR	- The use of IVUS was associated with a smaller reduced LLL (0.28 ± 0.48 mm vs. 0.46 ± 0.68 mm, *p* = 0.025) than angiography-guided PCI - There were no significant differences in the incidence of MACE in the two groups (18.3% vs. 22.6%, *p* = 0.513) - The event rate in the two groups for the secondary endpoints was similar apart from the incidence of ST that was lower in the IVUS arm (0.9% vs. 6.1%, *p* = 0.043)
Tan et al. [59]	IVUS vs. angiography-guided PCI	123 patients randomized at 1:1 ratio	24	LMS disease was defined as ≥50% stenosis by visual assessment	- MACE, was defined as death, non-fatal MI, and TLR-Safety endpoint was ST	- The overall MACE rate was lower in the IVUS-guided group than in the control group (12.8% vs. 27.3%, *p* = 0.049) - The ST rate was similar in the two groups (1.6% vs. 3.2%, *p* = 0.568)
IVUS-XPL [61]	IVUS vs. angiography	1400 patients randomized at 1:1 ratio	12	Long lesions ≥ 28 mm	- MACE, including cardiac death, TV-MI, or ID-TLR	- The MACE rate was lower in the IVUS arm compared to the angiography arm (2.9% vs. 5.8%, *p* = 0.007), mainly due a reduced ID-TLR (2.5% vs. 5.0%, *p* = 0.02)
ULTIMATE [63]	IVUS vs. angiography-guided PCI	1448 patients randomized at 1:1 ratio	12	All lesions deemed suitable for PCI	- Primary: TVF, including CD, TV-MI, and clinically driven TVR - Secondary: All-cause death, MI, TLR, ISR, stroke, and each individual component of the primary endpoint	- TVF occurred more often in the angiography than the IVUS-guided groups (5.4% vs. 2.9%, *p* = 0.019) - There was no difference in the outcomes for the secondary endpoint analyses
Liu et al. [64]	IVUS vs. angiography-guided PCI	336 patients randomized at 1:1 ratio	12	LMS lesions planned PCI	- Primary: MACE, including CD, MI, and TVR - Safety endpoint: the incidence of ST	- IVUS use was associated with a lower MACE rate than angiography-guided PCI (13.2% vs. 21.9%, *p* = 0.031) -The incidence of ST was similar in the two groups
RENOVATECOMPLEX-PCI [70]	Intravascular imaging- (IVUS or OCT) vs. angiography-guided PCI	1639 patients randomized at 2:1 ratio in the intravascular imaging and angiography-guided revascularization	25	True bifurcation lesions with side branches ≥ 2.5 mm, CTO’s, LMS, long lesions ≥ 38 mm, multivessel PCI, ≥3 stents, ISR, severe calcification, or ostial lesions	- Primary: TVF, defined as the composite of CD, TV-MI, or clinically driven TVR - Secondary: The individual components of the primary endpoint, TVF without procedure-related MI, a composite of TV-MI or CD, and definite ST	- TVF occurred in 7.7% of the patients in the intravascular imaging group and in 12.3% in the angiography group (*p* = 0.008) - The incidence of TVF without procedure-related MI was lower in the intravascular imaging group than in the angiography group (5.1% vs. 8.7%)
GUIDE-DES [72]	QCA vs. IVUS-guided revascularization	1528 patients randomized at 1:1 ratio	12	Any obstructive lesion listed for PCI	- Primary: TLF, defined as a composite of CD, TV-MI, or ID-TVR Secondary: All-cause death, MI, definite or probable ST, stroke, TLR and any revascularisation	- The incidence of TLF was similar in the QCA and IVUS-guided group (3.81% vs. 3.80%) (*p* = 0.99). - Secondary endpoint events were infrequent and similar in both groups
Bendary et al. [65]	IVUS vs. angiography-guided revascularization	181 patients randomized at 1:1 ratio	12	LMS lesions scheduled for DES implantation	- MACE, which encompasses CD, MI and TVR -Safety endpoint: ST	- Patients who underwent IVUS demonstrated a significantly lower MACE (3.3% vs. 18.7%, *p* < 0.001) than those who underwent the conventional method - There was no difference in MI, TVR, ST or in-hospital mortality rates between the groups (*p* > 0.05 for all)
IVUS-ACS [71]	IVUS vs. angiography-guided revascularization	3504 patients randomized at 1:1 ratio	12	Any culprit lesion in patients with an ACS treated with PCI using DES	- Primary: TVF, a composite of CD, TV-MI, or clinically driven TVR - Secondary: The individual components of the primary endpoint, TVF without procedural MI, TLR, bleeding and ST	- The TVF at 1 year occurred more often in the angiography than the IVUS arm (7.3% vs. 4.0%, *p* = 0·0001) - The incidence of TVF without procedural MI was also lower in the IVUS group (5.1% vs. 2.2%, *p* < 0·0001)

Footnote: ACS, acute coronary syndrome; CD, cardiac death; CTO, chronic total occlusion; DES, drug-eluting stent; ID-TLR, ischemia-driven target-lesion revascularization; ID-TVR = ischemia-driven target-vessel revascularization; ISR = in-stent restenosis; IVUS, intravascular ultrasound; LMS, left main stem; LLL, late lumen loss; MACE, major adverse cardiovascular events; MI, myocardial infarction; MLD, minimum lumen diameter; PCI, percutaneous coronary intervention; ST, stent thrombosis; TLR, target-lesion revascularization; TVF, target-vessel failure; TV-MI, target-vessel myocardial infarction; TVR, target-vessel revascularization.

**Table 2:** RCTs examining the prognostic value of OCT in guiding PCI over angiography.

Study	Studied Groups	Number of Patients	Follow-Up (Months)	Lesion Type	Endpoints	Results
DOCTORS [73]	OCT vs. Angiography-guided PCI	240 patients randomized at 1:1 ratio	6	Any lesion suitable for PCI	- Post-procedural FFR - Secondary: procedural complications	- OCT-guided PCI led to higher post-PCI FFR than angiography (0.94 ± 0.04 vs. 0.92 ± 0.05, *p* = 0.005) - There was no difference in the procedural complications between groups
Kala et al. [81]	OCT vs. Angiography-guided PCI	201 patients randomized at ~1:1 ratio	9	Culprit lesions in patients with a STEMI and reference diameter: 2.5–3.75 mm	- Primary: MACE; including death, MI and TLR - Secondary: In-segment area stenosis post PCI	- The MACE rate was similar in the two groups (3% vs. 1%, *p* = 0.623) - The in-segment area of stenosis was smaller in the OCT arm (6% vs. 18%, *p* = 0.0002)
OCTOBER [80]	OCT vs. Angiography-guided PCI	1201 patients randomized at 1:1 ratio	24	Bifurcation lesions with ≥50% stenosis in both the main (≥2.75 mm reference diameter) and side branch (≥2.5 mm reference diameter)	- Primary: MACE, defined as CD, TLMI, or ID-TLR - Secondary: CD, TLMI, TLR, a bifurcation lesion–oriented composite endpoint of CD, TLMI; TLR and a patient-oriented composite endpoint of death from any cause, MI, any coronary revascularization, or stroke	- MACE occurred in 10.1% in the OCT and in 14.1% in the angiography-guided PCI (*p* = 0.035) - There was no statistically significant difference in the outcomes for the secondary endpoints
ILUMEN IV [19]	OCT- vs. Angiography-guided PCI	2487 patients randomized at 1:1 ratio	24	Patients with diabetes and high-risk lesions including lesions causing a MI, long or multiple lesions (>28 mm stent), bifurcations needing two stents, severe calcification, CTOs and diffuse/multifocal ISR	- Primary imaging: MSA post PCI assessed by OCT - Primary clinical: TVF, defined as CD, TV-MI, or ID-TVR - The major secondary endpoint was target-vessel failure, excluding periprocedural myocardial infarction	- The MSA post PCI was larger in the OCT than the angiography-guided group (5.72 ± 2.04 mm^2^ vs. 5.36 ± 1.87 mm^2^, *p* < 0.001) - The TVF rate was similar in the two groups (7.4% in the OCT and 8.2% in the angiography arm, *p* = 0.45)
OCCUPI [79]	OCT vs. Angiography-guided-PCI	1604 patients randomized at 1:1 ratio	12	Culprit lesions in ACS, CTO, long lesions (≥28 mm), calcified, bifurcation lesions, LMS, small vessels (<2.5 mm), visible thrombus, stent thrombosis, ISR, or bypass graft lesions	- Primary: MACE, a composite of CD, MI, ST, or ID-TVR Secondary: Any revascularisation, TLR, periprocedural MI, stroke, bleeding events, rate of stent optimization confirmed by post-stenting OCT and CIN	- The primary endpoint occurred in 5% in the OCT and in 7% in the angiography-guided group (*p* = 0·023) - Any revascularization rate was smaller in the OCT group (6% vs. 2% *p* = 0.0009). There was no significant difference between groups for the other secondary endpoints

Footnote: ACS, acute coronary syndrome; CD, cardiac death; CIN, contrast-induced nephropathy; CTO, chronic total occlusion; DES, drug-eluting stent; FFR, fractional flow reserve; ID-TLR, ischemia-driven target-lesion revascularization; ID-TVR = ischemia-driven target-vessel revascularization; ISR = in-stent restenosis; LMS, left main stem; MACE, major adverse cardiovascular events; MI, myocardial infarction; MSA, minimum stent area; OCT, optical coherence tomography; PCI, percutaneous coronary intervention; ST, stent thrombosis; STEMI, ST-segment elevation myocardial infarction; TL-MI, target-lesion myocardial infarction; TLR, target-lesion revascularization; TVF, target-vessel failure; TV-MI, target-vessel myocardial infarction; TVR, target-vessel revascularization.

**Table 3:** RCTs comparing outcomes in patients having revascularization under IVUS and OCT guidance.

Study	Studied Groups	Number of Patients	Follow-Up (Months)	Lesion Type	Endpoints	Results
ILUMEN III: OPTIMIZE PCI [75]	IVUS vs. OCT vs. Angiography	450 patients randomized at 1:1:1 ratio	12	De novo lesions with a reference vessel diameter 2.25–3.50 mm and length < 40 mm	- Primary efficacy endpoint: post-PCI MSA - Primary safety endpoint: procedural MACE	- The MSA was comparable in the three groups (5.79 mm^2^ in the OCT, 5.89 mm^2^ in the IVUS, and 5.49 mm^2^ in the angiography arm) - There were no differences in procedural MACE in the three groups (*p* = 0.37)
OPINION [82]	IVUS vs. OCT-guided revascularization	829 patients randomized at 1:1 ratio	12	De novo lesions with diameter ≥ 2.5 mm listed for PCI	- Primary: TVF, defined as a composite of CD, TVR-MI and ID-TVR - Secondary: CD, MI, TVR-MI, ID-TVR and ID-TLR, MACE including CD, MI, or ID-TLR, ST, RS, stroke and CIN	- The TVF rate was similar in the two groups (5.2% vs. 4.9%, *p* = 0.042 for non-inferiority) - There was no difference between groups for all the secondary endpoints
iSIGHT [76]	IVUS vs. OCT vs. Angiography-guided PCI	156 patients randomized at 1:1:1 ratio	12	De novo lesions with a reference between 2.25 and 4.0 mm	Primary: Stent expansion rate in the IVUS and OCT groups Secondary endpoint: Stent expansion in the three groups	- Stent expansion was similar in the OCT and IVUS groups (98.01 ± 16.14% vs. 91.69 ± 15.75%, *p*_non-inferiority_ < 0.001) - Patients undergoing OCT-guided PCI had a higher stent expansion rate than those listed for angiography-guided PCI (90.53 ± 14.84%, *p* = 0.041), but there was no difference between IVUS and angiography-guided groups (*p* = 0.067)
OCTIVUS [83]	IVUS vs. OCT-guided PCI	2008 patients randomized at 1:1 ratio	12	The various types of significant coronary artery lesions (multivessel, left main vessel, bifurcations, extensive long coronary artery lesions) except for ST-segment elevation MI lesions	- Primary: TVF, defined as CD, TV-MI, or ID-TVR - Secondary: individual components of the primary endpoint, TLF, ST, stroke, repeat PCI, any rehospitalisation, bleeding, CIN, procedural complications and angiographic and imaging device success	- The primary endpoint had occurred in 2.5% in the OCT and in 3.1% in the IVUS-guided PCI groups (*p*_non-inferiority_ < 0.001) - There was no difference between IVUS and OCT guided revascularization in all the secondary endpoints

Footnote: CD, cardiac death; CIN, contrast-induced nephropathy; CTO, chronic total occlusion; DES, drug-eluting stent; ID-TLR, ischemia-driven target-lesion revascularization; ID-TVR = ischemia-driven target-vessel revascularization; ISR = in-stent restenosis; IVUS, intravascular ultrasound; LMS, left main stem; MACE, major adverse cardiovascular events; MI, myocardial infarction; MSA, minimum stent area; OCT, optical coherence tomography; PCI, percutaneous coronary intervention; ST, stent thrombosis; STEMI, ST-segment elevation myocardial infarction; TL-MI, target-lesion myocardial infarction; TLR, target-lesion revascularization; TVF, target-vessel failure; TV-MI, target-vessel myocardial infarction; TVR, target-vessel revascularization.

## Data Availability

No new data were created or analyzed in this study. Data sharing is not applicable to this article.
